# Characterization of an In-Situ Soil Organic Carbon (SOC) via a Smart-Electrochemical Sensing Approach

**DOI:** 10.3390/s24041153

**Published:** 2024-02-09

**Authors:** Vikram Narayanan Dhamu, Anil C Somenahally, Anirban Paul, Sriram Muthukumar, Shalini Prasad

**Affiliations:** 1 Department of Bioengineering, University of Texas at Dallas, Richardson, TX 75080, USA; vikramnarayanan.dhamu@utdallas.edu (V.N.D.); anirban.paul@utdallas.edu (A.P.); 2 Department of Soil and Crop Sciences, Texas A&M AgriLife Research, Overton, TX 75684, USA; anil.somenahally@ag.tamu.edu; 3 EnLiSense LLC, Allen, TX 75013, USA; sriramm@enlisense.com

**Keywords:** soil organic carbon, electrochemical sensing, in-soil measurement

## Abstract

Soil is a vital component of the ecosystem that drives the holistic homeostasis of the environment. Directly, soil quality and health by means of sufficient levels of soil nutrients are required for sustainable agricultural practices for ideal crop yield. Among these groups of nutrients, soil carbon is a factor which has a dominating effect on greenhouse carbon phenomena and thereby the climate change rate and its influence on the planet. It influences the fertility of soil and other conditions like enriched nutrient cycling and water retention that forms the basis for modern ‘regenerative agriculture’. Implementation of soil sensors would be fundamentally beneficial to characterize the soil parameters in a local as well as global environmental impact standpoint, and electrochemistry as a transduction mode is very apt due to its feasibility and ease of applicability. Organic Matter present in soil (SOM) changes the electroanalytical behavior of moieties present that are carbon-derived. Hence, an electrochemical-based ‘bottom-up’ approach is evaluated in this study to track soil organic carbon (SOC). As part of this setup, soil as a solid-phase electrolyte as in a standard electrochemical cell and electrode probes functionalized with correlated ionic species on top of the metalized electrodes are utilized. The surficial interface is biased using a square pulsed charge, thereby studying the effect of the polar current as a function of the SOC profile. The sensor formulation composite used is such that materials have higher capacity to interact with organic carbon pools in soil. The proposed sensor platform is then compared against the standard combustion method for SOC analysis and its merit is evaluated as a potential in situ, on-demand electrochemical soil analysis platform.

## 1. Introduction

Total carbon (TC) in soils exists in both organic and inorganic forms. All organic forms of carbon encompassing humic substances and many other organic moieties in the soil profile, but excluding larger organic compounds such as plant biomass residue and roots, are labeled as soil organic carbon (SOC). All inorganic forms of carbon, mostly comprising of soil carbonates, are called soil inorganic carbon (SIC). Conserving and increasing SOC stocks in agriculture soils is critical for sustainability of food production as increasing SOC stocks have been noted to enhance beneficial soil properties such as soil fertility, water and nutrient retention and crop yields [1]. Moreover, increasing SOC accumulation in agricultural soils is important for climate mitigation, as significant amount of atmospheric CO_2_ can be channeled into stable SOC pools for long-term storage [2]. Thus, SOC is considered an important soil health property [3]. Quantity of SOC stocks is a function of inherent soil properties, climatic conditions and land use management [4,5]. Many soil properties such as soil pH, soil depth, texture and parent material are also major drivers of SOC storage [6]. Additionally, land management practices such as crop diversity, tillage and grazing have been noted to significantly impact SOC stocks [6,7]. As a result, SOC concentrations are highly dynamic and vary significantly among different soil types and between agricultural systems [8]. Accurately estimating SOC concentrations in agricultural soil is important for assessing current stocks and making consequential changes to land use management to increase their stocks.

Increasing soil carbon stocks (SCS) is critical for sustainable agriculture production and mitigation of climate change impacts. Many complex interactions governed by climatic factors, soil characteristics and agronomic practices impact soil carbon pools; hence, it is a challenge to accurately assess and track changes in SCS in farmlands. Spatiotemporal variations of SCS within a soil profile are also important, as surface soil (within root-zone) and subsoil (below root-zone) stocks are differently sensitive to land use management and climate change effects. Soil carbon is represented in both organic and inorganic forms, and both pools are impacted by biogeochemical processes. Microbial transformation of organic matter to microbial metabolites and microbial biomass-associated organic forms are key process for preserving soil carbon in soil aggregates and mineral phases. However, microbial processes are also responsible for depleting several organic and inorganic soil carbon pools. These subtle changes in both carbon pools must be accurately accounted to subsequently understand the carbon sequestration potential. Moreover, concurrent assessment of soil bulk density is essential for determining SCS changes on a real basis. Current laboratory-based assessment of soil samples can only provide a snapshot assessment and are not feasible for large scale assessments. Field-based methods using spectroscopy and imaging techniques are less sensitive and suffer from interferences and are not suitable for subsoil assessment. These drawbacks have also hindered accumulation of large-scale datasets for improving carbon cycling models. In this work, we put together a sensor-based ‘bottom-up’ approach towards quantification of soil organic carbon (SOC) in an on-demand and field-deployment capable manner.

Current standard protocols for assessing SOC in agriculture fields include a number of laboratory assessment methods for SOC concentration or in situ proximal sensing techniques [9]. Widely used laboratory techniques are either based on chemical or thermal oxidation of SOC [10]. Thermal oxidation using dry combustion is currently the most reliable and accurate method for determining TC. Dry combustion at higher temperature (around 1000 °C) ensures oxidation of all TC to CO_2_ and subsequently an infra-red detector is used for accurately quantifying the amount of CO_2_ [11,12,13]. As a result, the dry combustion method using carbon analyzer equipment has been widely adopted as a reference method to compare or calibrate other methods of soil carbon analysis [11,14,15]. Alternatively, chemical oxidation and trapping of CO_2_ techniques such as the Walkley–Black method are also widely used as they are inexpensive and do not require a costly equipment [15]. However, chemical oxidation is less accurate due to incomplete oxidation of SOC and SIC in some soils and thus quantity of SOC recovery varies with soil type and nature of soil organic matter present [16]. Thus, chemical oxidation-based SOC estimation generally requires a correction factor to adjust for incomplete oxidation of SOC [17]. However, there is no consensus as different correction factors are used by researchers [15]. 

Accuracy of thermal oxidation-based SOC estimation is dependent on precisely separating SIC and SOC pools from TC. Samples are generally acid treated for removing carbonates [18]. Several acid pretreatment techniques are used and their efficacy for removing carbonates is variable depending on soil properties and type of carbonates [19]. For example, less concentrated HCL treatment may not completely remove all carbonates [20], particularly in high pH soils with higher SIC content [21], whereas strong acid mixtures may also oxidize some SOC pools [22]. There are no standard techniques to assess these tradeoffs and correct for biases under different acid treatment techniques. Accuracy of laboratory assessment for SOC is also dependent on obtaining a representative set of soil samples covering the variability of a field. However, soil sampling protocols are not uniformly implemented [23], particularly when farmers are not trained on sample collection. Several reports showed implicit sampling bias as a major source of error in estimation of SOC [24,25].

A common method considered for in-field trials for SOC determination is based on Near Infrared (NIR) spectroscopy works by using broad-spectrum light input between 780–2500 nm and probing the absorption and scattering aspects of the light at the reference and resonant frequencies to extract chemical compositional information about the sample probed. Since it utilizes an optical input to probe soil composition, the depth of sampling/testing using vis-NIR technique is limited to surface soil only unless fiber optic-based vis-NIR system is applied [26]. While this utilization exists in the scientific literature, the practical implications of visible-near-infrared reflectance (vis-NIR) spectroscopy to anticipate SOC substance and the application of this strategy at a larger scale remain challenging due to the tall spatial heterogeneity of SOC and the spatially subordinate connections of soil spectra and SOC content [27]. Another important factor to note is that this vis-NIR method is subjective and prone to errors with soil particle size and moisture levels, i.e., the predictive capability is variable regarding moisture present in the system and the particle size of soil [28,29]. Various pre/post-analysis spectrum treatments and complex model integrations including partial least squares regression, multiple linear regression and principal component analysis are required to boost system capability to predict SOC [30]. 

The sensor platform described herein is viable as a complementary methodology to standard laboratory protocols and will be beneficial for in situ measurement and real-time assessment of SOC in soils. The techniques used in this work to facilitate and report the carbon building in soil are based on pulsed voltammetric techniques [31,32,33], and, in particular, Square Wave Voltammetry (SWV) [34,35,36]. 

**Figure 1:**
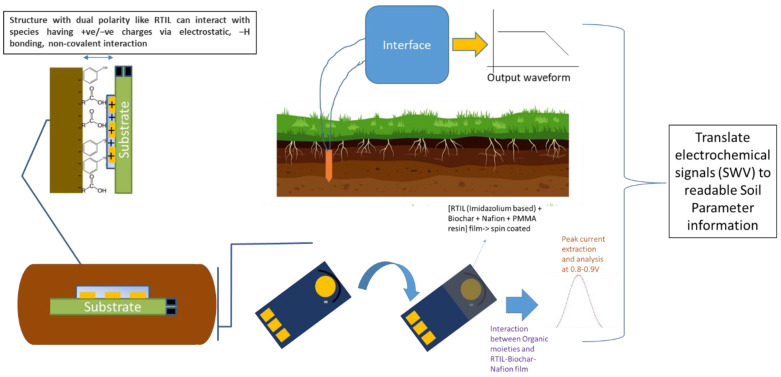
Schematic description of ‘bottom-up’ sensor approach to dynamically track and detect soil organic carbon (SOC).

An electroanalytical approach is to be evaluated in this study to track and manage soil organic carbon. As part of this setup shown in Figure 1, soil is used as a solid-phase electrolyte under probing in a manner similar to a standard electrochemical cell and the electrode probes are used to apply an input bias to facilitate breakdown of soil and, thereby, separate carbon from the soil matrix. This separated carbon would cause an electrochemical charge modulation at the electrode interface and can be quantified using appropriate modalities. Charge transfer and charge-withholding capacities of soil would then be investigated to truly understand the nature of soil’s electrochemistry. Hence, it is via this electrochemical kinetics that it is possible to quantify and monitor changes to soil organic carbon (SOC).

## 2. Materials and Methods

### 2.1. Sensor Preparation

The sensor system used for SOC sensor was based on a three-electrode FR-4 PCB design with a gold working electrode (WE) ENIG (electroless nickel immersion gold) finish, carbon paste counter electrode (CE) and silver/silver chloride reference electrode (RE) [37]. A composite element was developed to coat onto the sensor working electrode area; 100 µL of 1-Butyl-3-methylimidazolium tetrafluoroborate (BMIM BF4) was taken in a 95% *w*/*v* formulation to which 40 mg of activated Biochar in completely ground/powdered form was added. Then, 10 mg of Supelite DAX-8 was added to this mixture and mixed into a homogeneous composite. This composite was spin-coated onto the working electrode active area with 15 µL. This was then left to form a polymeric network onto the sensor over a period of hours. 

### 2.2. Sensor Testing

Next, to insert the sensor into the soil setup, a small crevice was created to insert the sensor into the ‘test well’ and soil–sensor interface was checked for good contact between functionalized sensor surface and soil matrix. The component used within this project for testing and measurement is the GAMRY potentiostat instrument (Warminster, PA, USA) for electrochemical analysis.

The test method applied for SOC sensor is described herein: input square wave pulsed voltage bias of step size 5 mV, pulse size 25 mV, frequency 25 Hz swept between voltage range 0–1.2 V. Then, the voltammogram output is recorded, wherein the peak current at the specific potential range 0.8–0.9 V is captured as a function of SOC modulation based on trial experiments (Depicted in Supplementary Information section). 

### 2.3. Soil Samples 

Ten soil samples were collected from farms representing a range of texture, pH and carbon stocks. Samples were collected from surface soil (0–20 cm). Soil samples were air dried, cleaned for large plant residues and then ground to pass through a 2 mm sieve. Soil samples were further sieved to a size of <0.15 mm to be used for SOC and SIC analysis. Samples were stored appropriately until further analysis. Soil sample texture was estimated using the feel method and soil pH was estimated in 1:1 saturated paste using a pH meter (Thermo Inc., Waltham, MA, USA). A subset of samples and their SOC concentrations were submitted to UTD for sensor training and calibration works. 

### 2.4. Estimating SOC Concentrations Using Laboratory Standard Method 

Standard protocols used for SOC and SIC analysis followed the details presented the book chapter [10]. Subsamples from <0.15 mm sieved soils were used for SOC and SIC analysis. Each soil sample was analyzed in five replicates. Samples were first analyzed for total carbon (TC) concentrations (mg/kg) using a dry combustion instrument equipped with infrared detector for CO_2_ quantification (Elementar Inc., Langenselbold, Hesse, Germany). Another set of subsamples were acid treated using 4 M HCl in combustion vessels until all reaction ceased. Samples were then air dried and analyzed for TC using the same dry combustion instrument. This analysis was considered as total SOC concentrations in mg/kg. Concentrations of SIC were obtained by subtracting SOC from TC. Concurrently, a subsample from both sets of air-dried soils used for TC and SOC analysis were oven dried to estimate soil moisture and correct for SOC and TC concentrations reported by the instrument. A known concentration of carbon-chemical standard was used with an individual set of ten samples. For additional quality check and control, one laboratory standard and one reference soil sample were used with each set. 

### 2.5. Statistical Analysis and Validation 

Ten sample results were used for final validation of sensor method for determining SOC concentration in mg/kg. Regression and Pearson correlation analysis were performed using the results obtained through reference method and sensor methods for SOC. Additionally, standard deviation analysis and percent difference between the reference method and sensor method were analyzed for comparing the reliability of the sensor method for determining SOC concentration. 

## 3. Results and Discussion

### 3.1. Electrochemical Sensor Methodology

Assessment of carbon as a parameter in soil samples in farm setting facilitates quantification and recording of its inherent physical and biochemical characteristics. Sufficient levels of soil nutrients are required for sustainable agricultural practices that typically exists as crop yield, pasture growth, etc. There are three basic types of soil, namely, sand, silt and clay, but in nature they exist as a combination of these different types (Figure S1). The cumulative charge of soil matrix is usually negative, and this affects the mobility of the organic and inorganic molecules in soil. Organic Matter present in soil (SOM) drives this negative charge due to presence of Carboxyl and phenol groups. Based on the properties of the various soil types, including but not limited to matrix structure (granular vs. cohesive as defined by OSHA regulation), pH, the inherent charge and hence the soil organic/inorganic matter concentration varies considerably. This therefore stipulates and drives the electrochemistry of the soil ecosystem.

### 3.2. Rationale behind Sensor Chemistry

#### 3.2.1. Chemical Composition of Soil Organic Carbon

Soil being the largest carbon reservoir on the planet implies that the anatomy of different carbon pools, especially within the organic matter section, is widely characterized and the dynamics of different specific functional groups of carbon in the soil are tracked to understand how carbon sequestration and soil quality changes over time [27].

There exists an active organic matter pool (AOM) or labile OC with different levels of activity, and can be found as these organic functional groups [38,39,40]:

1. Alkyl Carbon
2. O-Alkyl Carbon
3. Aliphatic Carbon
4. Aromatic groups
5. Carboxyl groups
6. Phenolic groups.

In addition to this, there exists a major chunk of organic carbon pools that are more stable and behave as sediments which do not break down or decompose over long period of time. These are denoted by the ‘umbrella’ term humic substances (HS) [41,42], which make up between 60–80% of total soil organic matter (SOM) [43] and contain compounds such as: Humic Acid (HA), Fulvic Acid (FA) and Humin. Therefore, in order to characterize the sensing mechanism between the sensor and the soil organic carbon targets, it was vital to deconvolute the constituents of SOC and determine how to create and optimize the sensor coating for selective SOC capture. The following section talks about the sensor and electrode characteristics that needed to be studied next prior to understanding the interaction between the sensor and soil OC functional blocks.

#### 3.2.2. Functionalized Electrode Characteristics

The sensor system herein consists of a rigid polymer probe with a three-electrode design overlay explained as follows and shown in Figure S3: Working electrode (WE) is the electrode region where we measure the signal activity of the soil matrix against the inherent reference electrode (RE). The third electrode is an auxiliary electrode (CE) that behaves as current source/sink and aids the working electrode resolution. 

Room-temperature ionic liquids (RTILs) are a combination of cationic and anionic molten salts, stable in room temperature, having exceptional thermal stability and negligible vapor pressure. Due to such unique properties, RTIL has been used for many applications in detection, extraction and synthesis in nanoparticles as a solvent, etc. Many RTILs possess good electrical conductivity and can operate in a wide electrochemical window. We are targeting those RTILs for this application in solid organic/organic compound sensing (soil OC) to be coated as a thin film on top of the electrode surface in the manner described in the methods section. In this regard, computation chemical modelling was utilized in this task to understand the geometry of the RTILs and its interactions with solid organic and inorganic compounds in soil such as various forms of carbon, especially within the organic pools. 

#### 3.2.3. Computational Chemistry Model of RTIL-Soil Organic Carbon Interactions

Gaussian software (Gaussian 16 and GausView6, Wallingford, CT, USA) with Hatree Fock and DFT calculation of ground state-optimized structure, energy (HOMO, LUMO), enthalpy, Gibbs free energy and bond distance was applied to understand the specific interaction, frequency of interest, etc., between the RTIL transducer-cum-support electrolyte and the soil electrolyte. Following the structure and binding interaction optimization, the Gaussian software is also used to simulate and plot the NMR, IR, Raman spectra and juxtapose it with our experimental results so as to understand how the RTIL [BMIM] [BF4] interacts with the different organic pools in soil.

**Figure 2:**
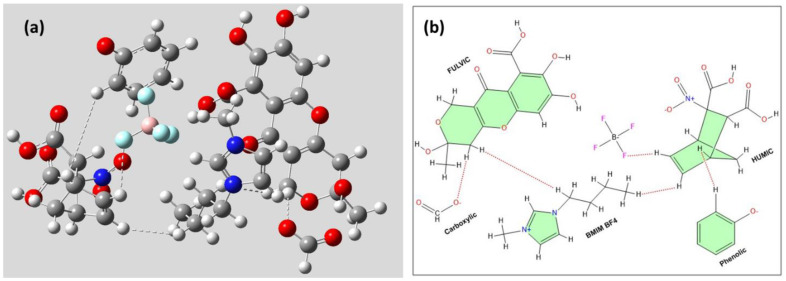
(**a**) Gaussian representation for the interaction of RTIL-HUM-AOM pool showing distinct non-covalent interactions; (**b**) chemical structural representation of the interaction for simplification, clearly depicts all the interactions.

A computational study has been done to visualize the interaction of the BMIMBF_4_ and Humic–Fulvic (HUM: Humous pool)/Phenolic-Carboxylic (AOM: Active Organic matter pool). For this purpose, BMIMBF_4_-Humic-Fulvic-Phenolic-Carboxylic has been optimized using Gaussian software, method: Hartree Fock, having a basis set of 6–31 g (d). The optimized structure of BMIMBF_4_-HUM-AOM is depicted in Figure 2a. The interaction is also graphically depicted in Figure 2b for better understanding. The optimized structure of BMIMBF_4_-HUM-AOM suggests that there is a strong ionic interaction between BMIM^+^-BF_4_^−^-HUM-AOM, which makes this composite species stable. The result suggests the presence of five strong non-covalent interactions. We have calculated the thermodynamic properties of BMIMBF_4_-HUM, BMIMBF_4_-HUM-AOM, depicted in Table 1. 

The result depicts a decrement of free energy of pristine RTIL by ~3× times, upon interaction with HUM pool, whereas a decrement of ~3500× times had been observed when AOM pool is introduced, which suggests the RTIL-HUM-AOM interaction is hugely favorable towards stability.

We have also calculated the HOMO-LUMO energy gap between BMIMBF4, BMIMBF_4_-HUM and BMIMBF_4_-HUM-AOM, and the graphical representation of HOMO-LUMO orbital is depicted in Figure S4a–c. All the result depict that the electron cloud is mostly situated close to the vicinity of the imidazole ring as it possesses higher µ-resonance.

Additionally, the HOMO-LUMO energy of [BMIM] BF_4_, [BMIM]BF_4_-HUM and [BMIM]BF_4_-HUM-AOM is depicted in Table S1. We have calculated the HOMO-LUMO energy gap between RTIL, RTIL-HUM and RTIL-HUM-AOM:

$$\begin{array}{l} \left(E_{HOMO}^{BMIMBF4}-E_{LUMO}^{BMIMBF4-HUM}\right)-(E_{LUMO}^{BMIMBF4}-E_{HOMO}^{BMIMBF4-HUM})\\ =\;(-0.40333\;+\;0.03128)\;-\;(-0.03525\;+\;0.33467)\\ =\;-0.37205\;-\;0.29942\\ =\;(-)0.67147\;\mathrm{Hartree}\\ \left(E_{HOMO}^{BMIMBF4}-E_{LUMO}^{BMIMBF4-HUM-AOM}\right)-(E_{LUMO}^{BMIMBF4}-E_{HOMO}^{BMIMBF4-HUM-AOM})\\ =\;(-0.40333\;+\;0.01846)\;-\;(-0.03525\;+\;0.61801)\\ =\;-0.38487\;-\;0.58276\\ =\;(-)0.96763\;\mathrm{Hartree}\\ (E_{HOMO}^{BMIMBF4}-E_{LUMO}^{BMIMBF4})=-0.40333+0.03525\\ =\;(-)0.36808\;\mathrm{Hartree} \end{array}$$

From the calculation, it has been found that the HOMO-LUMO energy gap has been substantially reduced to ~25% after the formation of the BMIMBF_4_-HUM complex, whereas there is a ~60% reduction of HOMO-LUMO energy gap upon formation of the RTIL-HUM-AOM complex. This suggests that the RTIL-HUM-AOM interaction is more feasible than RTIL-HUM and RTIL alone, which strongly suggests the hypothesis regarding the stability of the RTIL-HUM-AOM complex. 

We have also calculated the fundamental vibration pattern of the complex and the result is depicted with comparison to pristine RTIL as an absorbance output, depicted in Figure S5a–c. The result suggests no appearance of new compounds as no new peaks have been found. Moreover, most of the fingerprint peaks can be seen shifted, depicting the formation of a new complex but stabilized with non-covalent interactions. The fundamental FTIR absorption peaks of BMIMBF4, BMIMBF4-HUM and BMIMBF4-HUM-AOm have been depicted in Table 2 [39,44].

The result depicts presence of standard peaks present, such as at 1206 cm^−1^ for BMIMBF_4_ having standard -C-H bending for BMIM ring. The most important interaction in BMIMBF_4_ is the interaction between BMIM^+^ and BF4^−^ depicted by 2624 cm^−1^. The most interesting fact is that the interaction between BMIM^+^ and BF4^−^ is not there in other two complexes depicting the interaction of RTIL with SOC [45].

#### 3.2.4. Material Selection and Basis of Interactions

Biochar [46] is the leftover residue which is a type of charcoal produced by burning of biomass material in a pyrolytic environment that forms a carbon powder that has highly useful properties. When activated by either using reagents like acid, etc., or by using heat stimulation biochar, it can be ‘activated’, leading to highly electroactive behavior that makes it desirable for use in sensing applications. Different studies indicated that addition of biochar as an amendment to soil showed significant increase in the soils ability to sequester and hold more soil carbon of different pools, especially within the organic fraction. This included total C, organic C, microbial biomass C, labile C and fulvic acid, whose increase ranged from 20% to 85% [47]. Biochar was hence picked for this dual purpose due to being a good transducer material with good affinity for carbon–carbon (pi- bond) based interactions. Additionally, it was seen from the literature that RTILs act as a stabilizing agent and a dissolution medium that garners surfactant and solvatochromic properties causing other elements in its composition to form C–C bonds which is a vital physio-chemical interaction applicable in soil organic carbon capture and detection [48].

DAX-8 resin is a type of polymeric resin made of polymethyl methacrylate (PMMA) that has significant absorptive properties and affinity towards humic and fulvic acid. It was seen in different studies that were looking for separation characteristics of both aliphatic and humic carbon extracts with the use of DAX-8 resin. This showed that utilization of this resin within the composite-sensing material would boost the SOC detection strategy and the polymeric structure would further positively impact the structural fine chemistry of the composite-sensing ‘ink’ [49,50].

### 3.3. Sensor Characterization towards Soil Organic Carbon Quantification

The fundamental principle behind the approach is focused on the mechanism of organic carbon pools (SOC) in soil matrices interacting with the sensor system, becoming excited at a certain specific potential and causing a corresponding polar signal that is tracked and modeled with respect to concentration of SOC. 

Then, this electrochemical signal obtained as a function of the interfacial activity at the electrode is provided as an input into a reference model to determine an overall SOC quantification for the soil based on training and test data. The sensors used are functionalized with correlated ion surface treatments that interact chemically with target analytes in soil when a bias is applied. This interaction is transduced to an electrical output that varies from the baseline due to modulation of the electrochemical signal parameters at the interface. This is recorded and reported as percentage (%) change in SOC.

**Figure 3:**
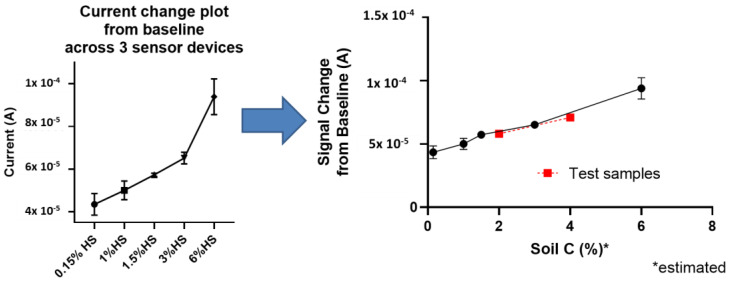
Consolidated data plots—Calibration profile in spiked soil and testing of calibration curve with 2 unique samples (represented in red).

The specific potential range extracted from the square wave voltammetry trials as described in the methods section is where the sensor surface is polarized and effectively interacts with the organic carbon pools in soil. So, the peak current signal at this potential is correlated to the SOC levels in the respective soil sample. Therein, a correlation function is modeled to build the calibration curve- using a soil mimic construct with minimal SOC. This was tested as a zero measure and its peak current acts as a baseline. Then, soils of different SOC levels are tested, and the peak current is measured. The SOC levels were varied by adding humic substances and other organic matter amendments to the soil and measured for reference. The difference between the peak current of the test soil sample and the soil baseline is plotted against SOC % to convolve the relationship/translation between the signal to SOC levels as depicted in Figure S7 (response observed and constructed in a KCl buffer in liquid phase) and Figure S7 representing the raw square wave voltammograms in soil as a solid-state electrolyte. Figure 3 (left) then shows the extracted current consolidated plot in soil media across triplicate sensor measurements which thereby serves as the calibration curve response for the soil organic carbon (SOC) sensor. 

### 3.4. Sensor Performance in Soil

The performance of the sensor calibration was tested with two standard samples (values ascertained from a standard method) whose signal characteristics were superimposed on the calibration curve to extract recovered SOC% values depicted in Figure 3 (right). This was then compared to the reference values to determine sensor viability towards sample testing.

### 3.5. Correlation Study: Soc Sensor vs. Lab Standard Method

#### 3.5.1. Properties of Soil Samples Used in This Study

Soil type, soil pH and average SOC and SIC concentrations for the soil samples used in this study are presented in Supplementary Table S2. Soil texture ranged from sandy loam (<10% clay) to clay texture (>45% clay). Soil pH ranged from 5.2 to 8.3. The concentration of SOC ranged from 0.1% to 3% and concentration of SIC ranged from 0.01% to 1.1%. The diversity of soil properties affirmed in this validation study encompasses a broad range of soil types. This selection strategically covers the anticipated variations in Soil Organic Carbon (SOC) levels found in agricultural soils globally.

#### 3.5.2. Comparison of SOC Concentrations Determined by Reference Method and Sensor Method

**Figure 4:**
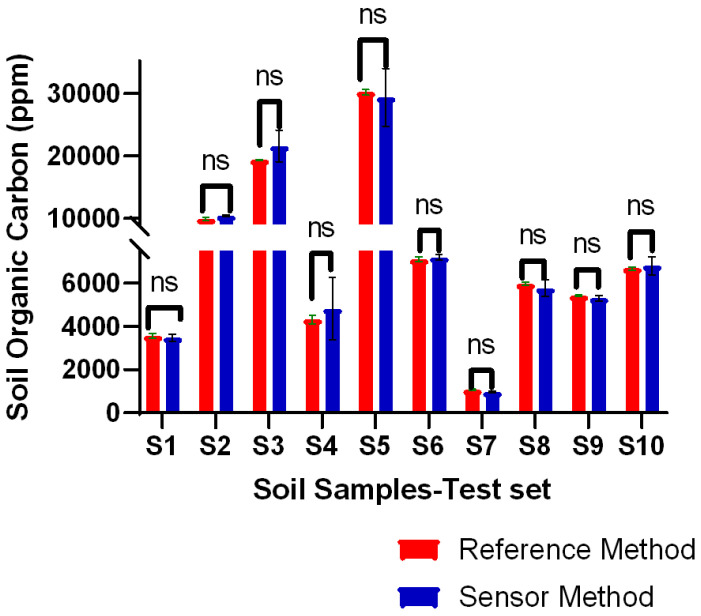
Competitive data results between sensor and standard reference methods of validation study with 10 field soil samples of different textures and different SOC ranges. (ns: not significant).

To determine the robustness of the sensor model and to quantitatively determine the efficacy metrics in terms of accuracy and precision against the standard in-lab practice, we conducted multiple measurements using three independent sensor chips to calibrate the organic carbon (SOC) response profile. These measurements were performed to encompass and add the sensor response and thereby intelligence from various soils with different levels of stable organic carbon pool compositions as represented by the texture and composition table in the supplementary section (Table S2). These value parameters, initially represented as %SOC, were converted to parts per million (ppm) by multiplying by a factor of 10,000 and cross-referencing with data obtained through combustion methods thereby obtaining training and test performance profile. Figure 4 shows the results for SOC concentrations obtained from both reference and sensor methods.

This train vs. test performance profile represented below as a bar plot was also depicted using a tabular column wherein, statistical significance (student’s *t*-test) was underscored and yielded that across all 10 soil samples, with a *p*-value greater than 0.05 there was no observable difference between the in-lab and in-soil sensor modalities. The overall aggregated difference value against N = 10 samples was 4.94%, which denotes a highly desirable < 5% against the lab standard analysis. The range of variance was observed between 1.36% to 10.2%, as detailed in Table S3. This cemented that the sensor’s robustness tested across a spectrum of soils with diverse textures and SOC concentrations was well within a sought-after scope, utilizing a benchtop electroanalytical quantitative device.

### 3.6. Blinded Study to Determine Feasibility of Sensor

To further cement the ability of the sensor to determine and quantify SOC levels in soil- N = 10 soil samples were obtained from a soil science collaborator and a fully blinded correlation study was conducted between the two teams (reference and sensor team). The results from the blinded study depicted in Table 3 showed that percent difference between the two methods ranged from 1.5% to 11.3%, demonstrating high degree of reliability of sensor method for determining SOC concentrations. Bivariate correlation and regression results for SOC concentrations between reference and sensor outputs are presented in Figure S8. There was a significant positive correlation (r = 0.9984, *p* < 0.05) for SOC concentrations reported by the reference and sensor outputs obtained from the Pearson’s correlation test. A smaller error percentage between the two methods and a significant positive linear relationship across the range of SOC concentrations demonstrated that sensor method was capable of reliably estimating SOC concentrations from all soil types included in this study.

## 4. Conclusions

It was concluded that the sensor method determined the SOC concentrations in mg/kg at an average of ≤5% and maximum variance < 12% reliability compared to the reference method. The sensor method showed potential to replace current standard practices of SOC estimation in laboratory based on extensive sampling and expensive equipment. 

Successful validation of these sensors in-field will increase adoption by stakeholders and real-time reporting of soil carbon stocks, which should enhance the integrity of soil carbon credit accounting and market confidence for trading soil carbon credits. Additionally, the proposed sensor system can be tagged and holds the potential as a smart sensor to be paired with computing platforms via a wired or wireless communication channel that can be utilized for long-term tracking, modeling and mapping geo-spatial organic carbon data.

## Figures and Tables

**Table 1:** Thermodynamic properties of BMIMBF_4_ and BMIMBF_4_-HUM-AOM.

Compound	EE+ Free Energy (Hartree)	EE+ Enthalpy (Hartree)
BMIMBF_4_	−0.387219	−0.317910
BMIMBF_4_-HUM	−0.928802	−0.747894
BMIMBF_4_-HUM-AOM	−3302.814	−3302.653

**Table 2:** FTIR peak description of the 3 different IR spectra: (1) RTIL only, (2) RTIL + Humic substances and (3) RTIL + Humic substances + Active organic matter pool.

Absorption Peaks (cm^−1^)		
BMIMBF_4_	BMIMBF_4_-HUM	BMIMBF_4_-HUM-AOM
**399** (B-F stretching)	**1174** (BMIMC-H bending)	**1275** (BMIM-C-H bending)
**758** (BMIM -C-H stretching asymmetric)	**1447** (Humic C-H bending)	**1784** (Fulvic-H-F of BF_4_)
**1206** (BMIM -C-H bending)	**1934** (-C=O stretching)	**1845** (Humic C=C)
**1232** (N-B interaction)	**3386** (Fulvic -C-H stretching)	**1948** (-C=O stretching)
**2624** (BMIM -C-H stretching symmetric)	**3549** (-O-H stretching)	**3044** (-C-H carboxylic)

**Table 3:** SOC levels correlation between reference and sensor methods for ten blind soil samples.

Sample ID	Reference Method (mg/kg)	Sensor Method (mg/kg)	% Difference between the Methods
T1	4438 (±339 ^¥^)	4341.6	2.2
T2	7100 (±107)	7346.0	3.5
T3	1072 (±42)	973.0	9.2
T4	19,273 (±166)	17,104.5	11.3
T5	9919 (±299)	9636.9	2.8
T6	6693 (±75)	7091.4	6
T7	30,228 (±542)	28,590.7	5.4
T8	4850 (±62)	4777.0	1.5
T9	5972 (±79)	5849.5	2.1
T10	5427 (±44)	5203.7	4.1

^¥^ values in parentheses are standard deviations.

## Data Availability

The datasets presented in this article are not readily available because the data is part of an ongoing study among other aspects. Requests to access the datasets should be directed to the corresponding author.

## Supplementary Materials

The following supporting information can be downloaded at: https://www.mdpi.com/article/10.3390/s24041153/s1.
