# Supplementary material for: Characterization of an In-Situ Soil Organic Carbon (SOC) via a Smart-Electrochemical Sensing Approach
Source: Sensors (Basel). 2024 Feb 9;24(4):1153. doi: 10.3390/s24041153 (PMC10892086; doi:10.3390/s24041153)
Supplement: Supplementary file 1 [file sensors-24-01153-s001.zip › sensors-2823822-supplementary.pdf]

Characterization of in-situ Soil Organic Carbon (SOC) via a Smart-Electrochemical Sensing approach

Vikram Narayanan Dhamu[1], Anil Somenahally[2], Anirban Paul[1], Sriram Muthukumar[3], Shalini Prasad[1,*]

[1]Department of Bioengineering, University of Texas at Dallas, Richardson, Texas 75080, USA

[2]Department of Soil and Crop Sciences, Texas A&M AgriLife Research, Overton, Texas 75684, USA

[3]EnLiSense LLC, Allen, Texas 75013, USA

*Corresponding email: shalini.prasad@utdallas.edu

Supplementary Information:

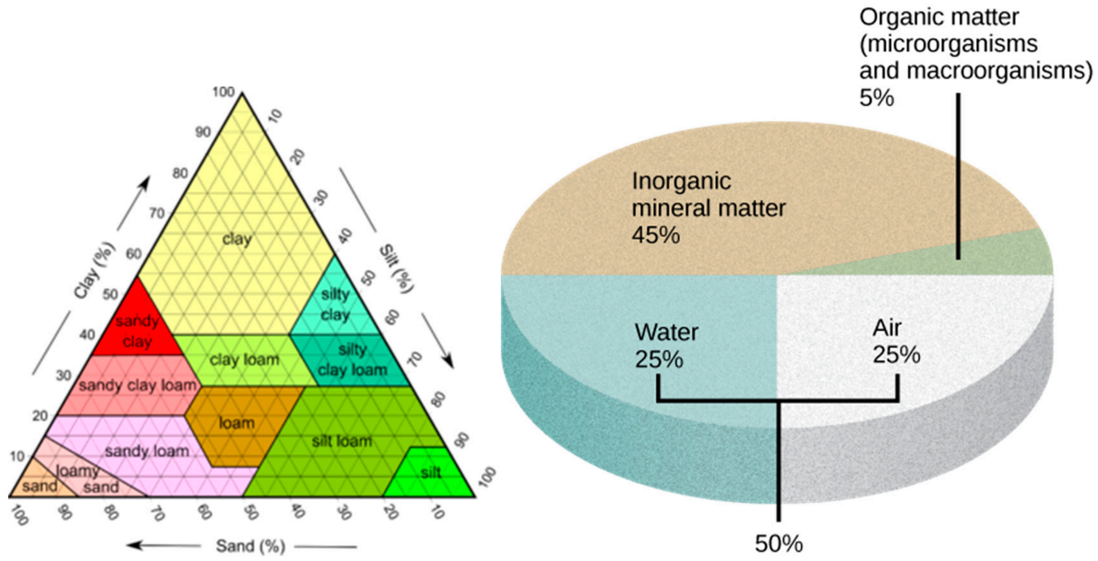

Figure S1. Soil Classification Chart and Composition of soil matrix. (Extracted from USDA repository)

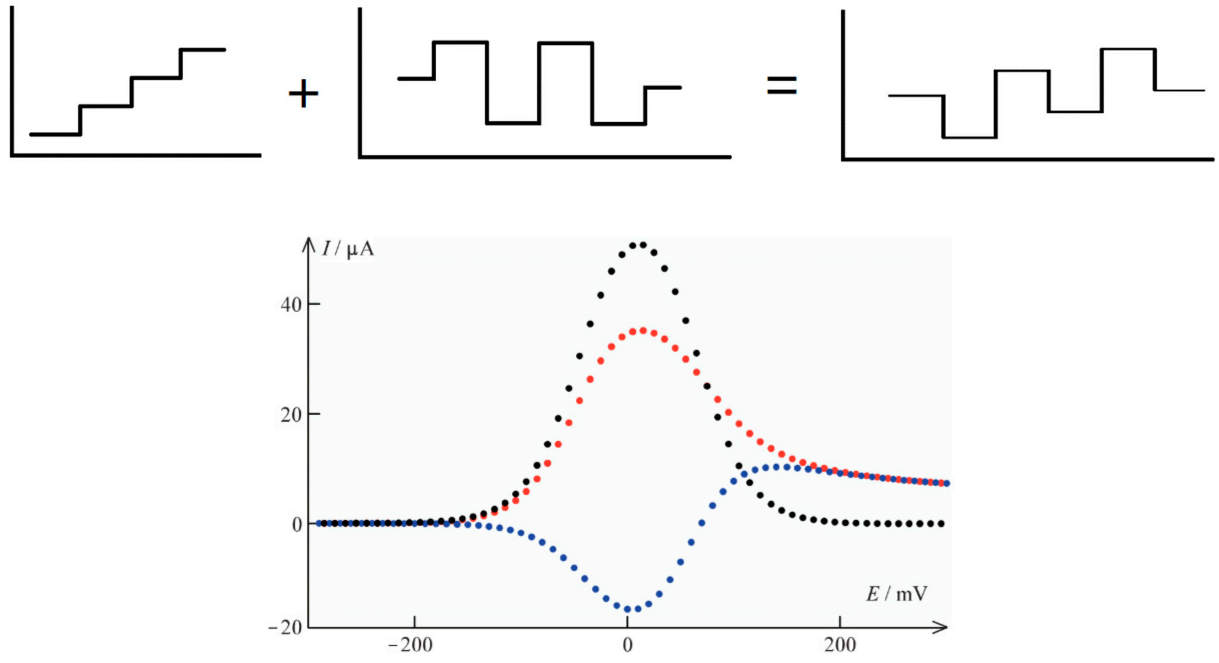

The forward, backward, and net square-wave voltammograms

Figure S2. (Top) Schematic description of voltage waveform for SWV. (Bottom) Square-Wave Voltammogram denoting all current outputs. (Extracted from Wikipedia and other open sources)

<u>Figure - Explanation:</u>

The techniques used in this work to facilitate and report the carbon building in soil are based on pulsed voltammetric techniques [31–33] such as: Square Wave Voltammetry (SWV) and Differential Pulse Voltammetry (DPV). DPV is a voltammetry method used to make electrochemical measurements where a series of regular voltage pulses is superimposed on the potential linear sweep or staircase pulse. The current is measured immediately before each potential change, and the current difference is plotted as a function of potential. SWV is another pulse voltammetry method similar to DPV that uses a combined square wave and staircase potential applied to a stationary electrode Figure S2 i.e., resultant potential waveform can be viewed as a superposition of a regular square wave onto an underlying staircase pulse. The current is sampled in this case at

two points - once at the end of the forward potential pulse and again at the end of the reverse potential pulse (in both cases immediately before the potential direction is reversed)

The current response in the case of SWV is a single peak determined by the difference between the forward and reverse current sampling. It is given by the following equation:

$$\Delta i_p = \frac{nFAD_0^{1/2}C_0^*}{(\pi t_p)^{1/2}}\Delta\Psi_p \quad \text{Eq (S1)}$$

Here, Δi_p is the differential current peak value, A is the surface area of the electrode, C_0^* is the concentration of the species, D_0 is the diffusivity of the species, t_p is the pulse width, and $\Delta\Psi_p$ is a dimensionless parameter which gauges the peak height in SWV compared to that of normal pulse voltammetry. An example of a square-wave voltammogram is given in the supplementary section (Figure S2).

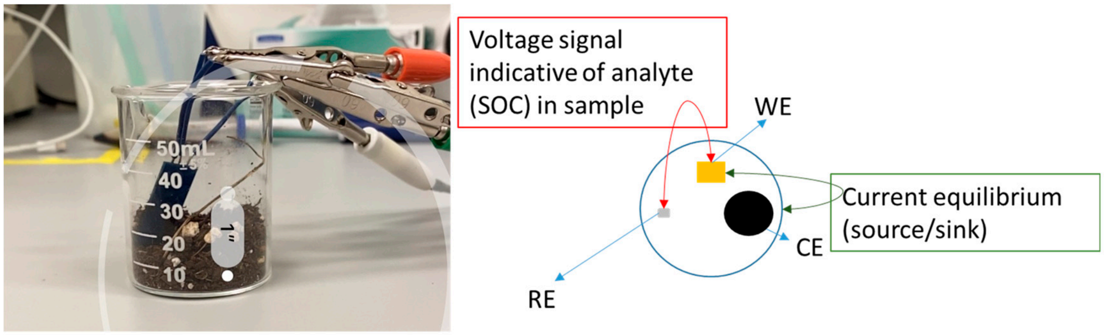

Figure S3. Experimental setup and electrode functional schematic representing sensor system.

Table S1: Calculated HOMO-LUMO energy of the RTIL, RTIL-HUM and RTIL-HUM-AOM

Compound	E_{HOMO} (Hartree)	E_{LUMO} (Hartree)
$BMIMBF_4$	-0.40333	-0.03525
$BMIMBF_4$-HUM	-0.33467	-0.03128
$BMIMBF_4$-HUM-AOM	-0.61801	-0.01846

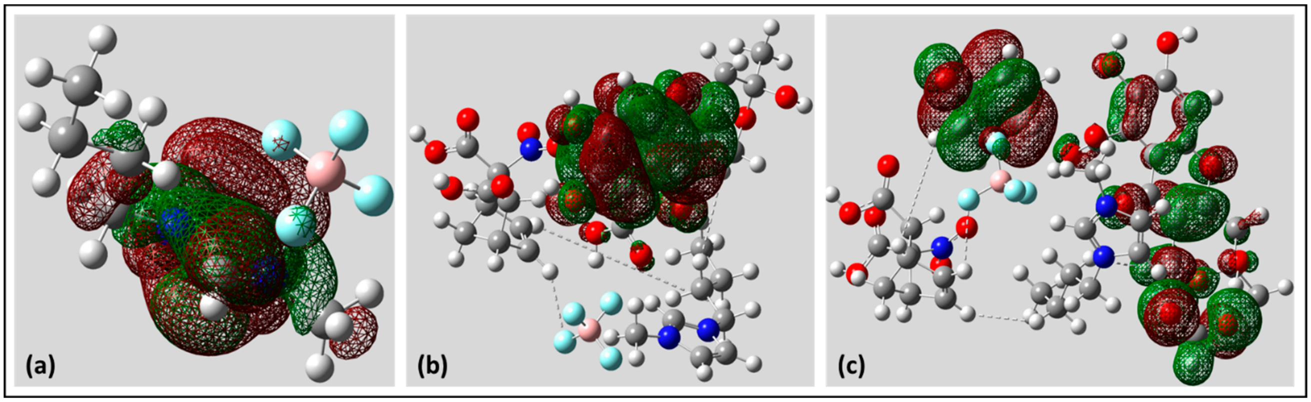

Figure S4. Schematic representation of HUMO-LUMO orbital between the RTIL and SOC interactions.

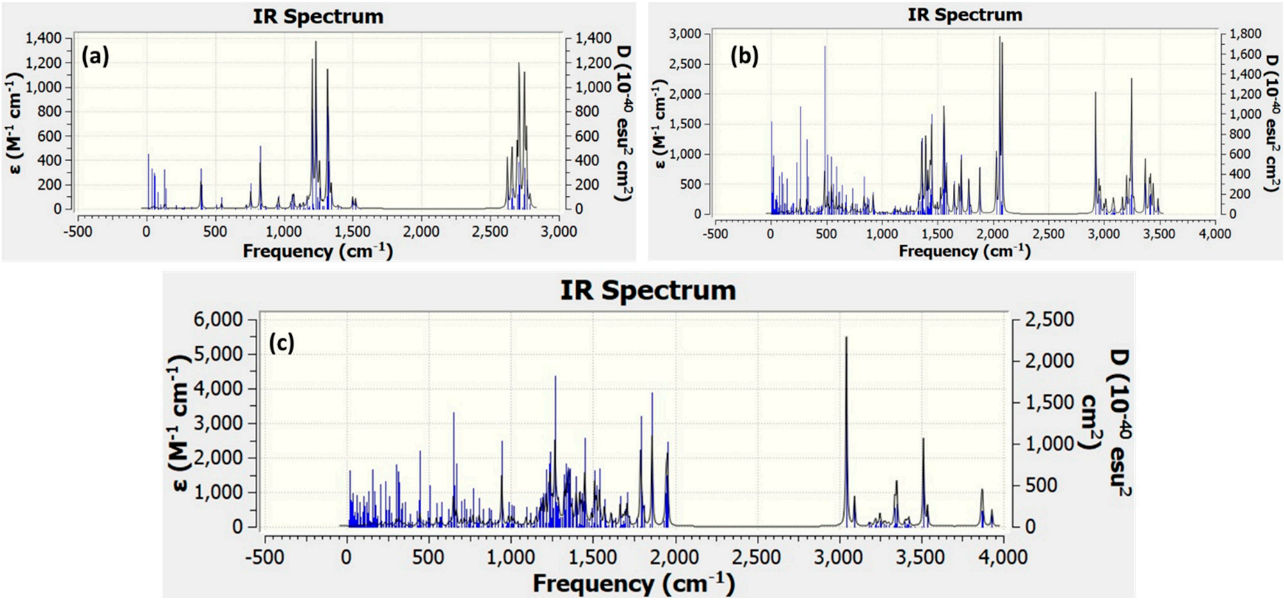

Figure S5. Computational IR spectra of A) RTIL only B) RTIL + Humic substances and C) RTIL + Humic substances + Active organic matter pool

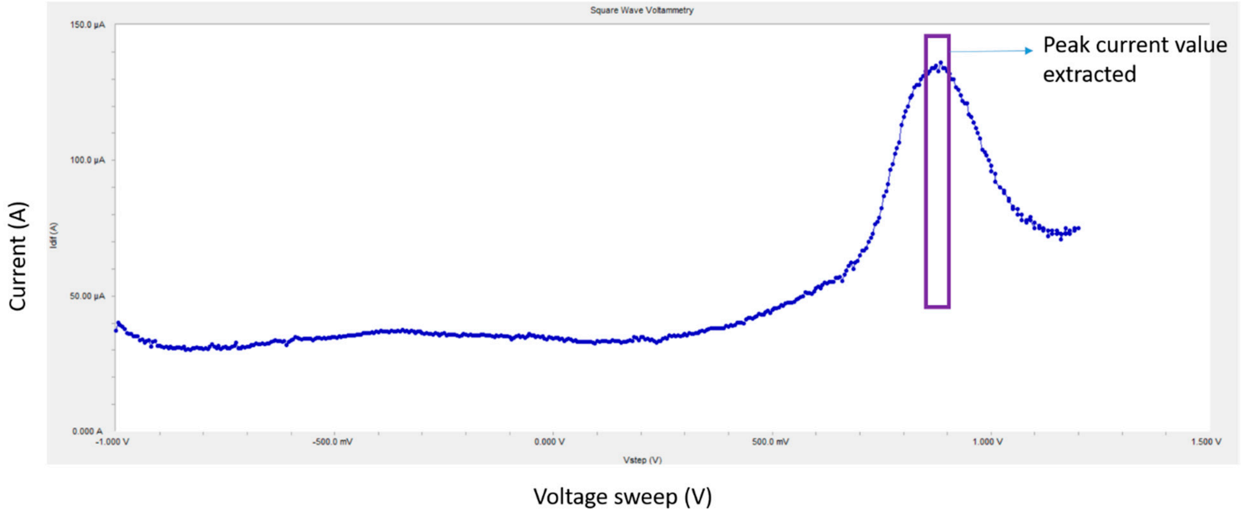

Figure S6. Depiction of the square wave voltammogram OUTPUT wherein- the peak current at the specific potential range 0.8-0.9V is captured as a function of SOC modulation.

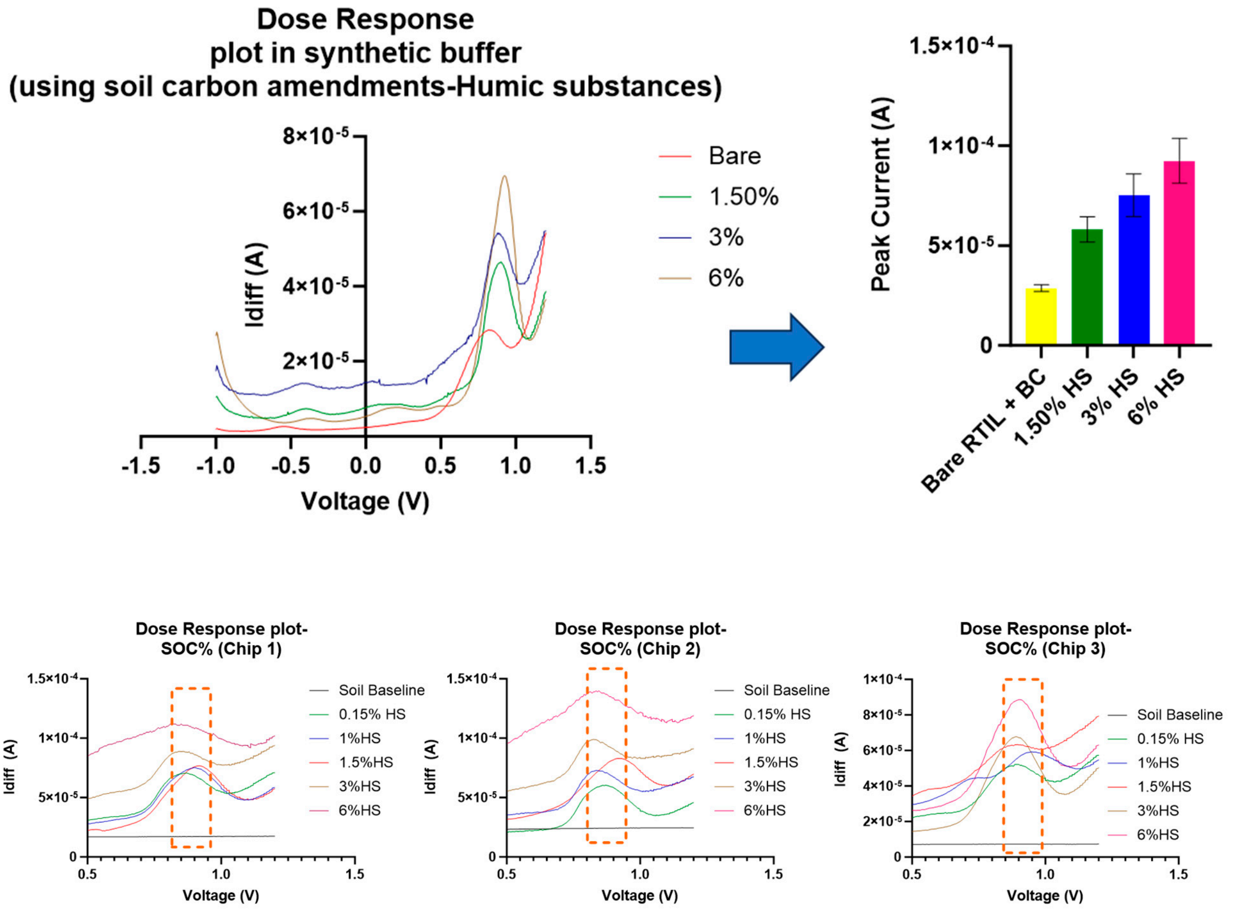

Figure S7. Test results of sensor stack response in (Top) 0.1M KCl buffer and (Bottom) soil phase (N=3 chips individual response)

Table S2. Soil properties of soil samples used for sensor validation.

Sample ID	Texture	pH	Approximate SOC (%)	Approximate SIC (%)
S1	Sandy loam	6.04	0.3	0.03
S2	Clay-loam	7.49	0.9	0.07
S3	Loam	6.72	1.9	0.08
S4	Loamy-clay	8.24	0.4	0.8
S5	Loam	7.26	3.0	0.3
S6	Clay	6.9	0.7	0.06
S7	Sandy	5.29	1.1	0.008
S8	Clay loam	8.19	0.6	0.08
S9	Loamy clay	8.29	0.5	1.1
S10	Clay loam	7.47	0.7	0.08

Table S3. Correlation of reference vs sensor method and difference between 2 methods using collected field soil samples. ((Final) Mean Variance across samples between methods: **4.941%)**

Sample#	Reference Method (ppm)	Sensor Method (ppm)	SD	Difference from reference method %
S1	3654	3481.107	159.85	4.73
S2	9919	10436.5	96.87	5.22
S3	19356	21095.02	2544.45	8.98
S4	4438	4822.89	1457.98	8.67
S5	30228	29409.27	4645.26	2.71
S6	7100	7196.8	132.83	1.36
S7	1072	962.4933	37.77	10.2
S8	5972	5768.753	382.13	3.4
S9	5427	5303.03	122.31	2.3
S10	6693	6816.112	411.91	1.84

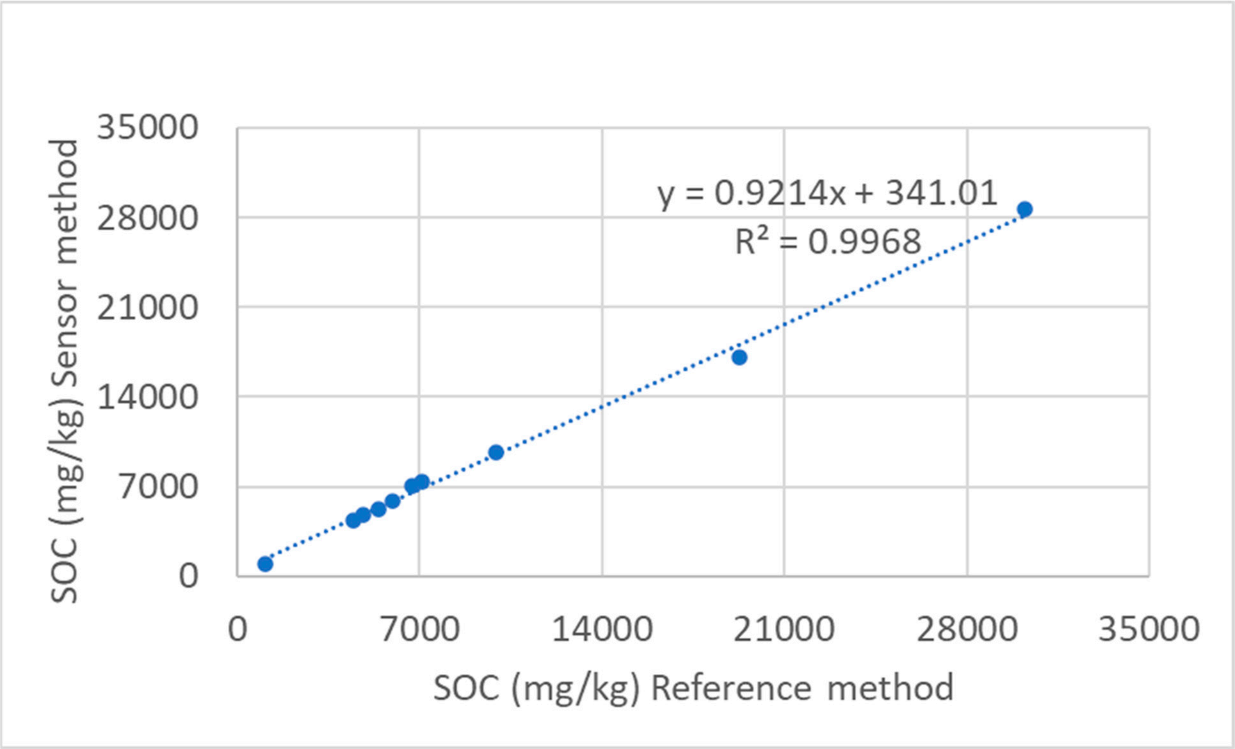

Figure S8. Correlation analysis of SOC concentrations obtained from reference and sensor analysis for the soil samples.

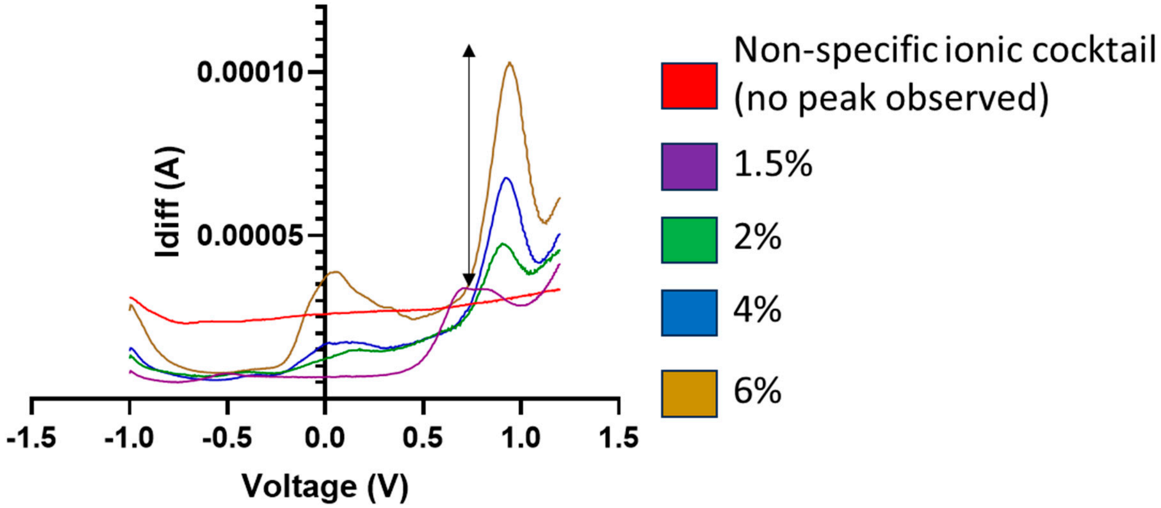

Figure S9. Specificity study results depicting sensor response for increase in SOC levels (increase in peak current) vs non-specific ionic cocktail containing NPK (nitrate, phosphorus, potassium) + chloride solutions where no peak is observed.

The current threshold due to noise (non-specific) factors has been considered and 1.96*SD (non-specific current) + non-specific current threshold has been considered as the SNR (signal to noise ratio) to calculate sensor performance parameters and LoD (limit of detection).
